# Seasonal Formation, Sources, and Long-Term Evolution of PM_2.5_-Bound Water-Soluble Inorganic Ions in Nanchang, China: The Increasing Importance of Wintertime Nitrate

**DOI:** 10.3390/toxics14090828

**Published:** 2026-09-18

**Authors:** Wei Guo, Zongzhong Ren, Junkang Wu, Kang Du, Lichen Deng, Yue Qian, Yong Luo, Cheng Liu, Yue Liang, Hao Zhao

**Affiliations:** 1 National Key Laboratory of Uranium Resources Exploration–Mining and Nuclear Remote Sensing, East China University of Technology, Nanchang 330013, China; liuc215@ecut.edu.cn (C.L.); liangyue@ecut.edu.cn (Y.L.); 2024210787@ecut.edu.cn (H.Z.); 2 Jiangxi Provincial Key Laboratory of Genesis and Remediation of Groundwater Pollution, School of Water Resources and Environmental Engineering, East China University of Technology, Nanchang 330013, China; 3 Nanchang Environmental Engineering Assessment Center, Nanchang 330038, China; ren13732920773@163.com (Z.R.); wjk522050082@163.com (J.W.); paihukang@163.com (K.D.); 4 Key Laboratory of Climate Change Risk and Meteorological Disaster Prevention of Jiangxi Province, Nanchang 330096, China; 18720068636@163.com (L.D.); qy_yue@126.com (Y.Q.); 5 Jiangxi Provincial Ecological Environment Monitoring Center, Nanchang 330039, China; u495294416@163.com; 6 State Environmental Protection Key Laboratory of Monitoring for Heavy Metal Pollutants, Changsha 410019, China

**Keywords:** secondary inorganic aerosol, atmospheric oxidation, positive matrix factorization, regional transport, multipollutant control, health relevance

## Abstract

Water-soluble inorganic ions (WSIIs) are major components of PM_2.5_, yet their seasonal characteristics, formation, sources, and long-term evolution in Nanchang remain poorly resolved. Daytime and nighttime PM_2.5_ samples collected in summer and winter were analyzed for nine WSIIs using statistical, source-apportionment, trajectory, fire-hotspot, and historical analyses. Mean WSII concentrations were 8.01 ± 3.58 μg m^−3^ in summer and 23.97 ± 9.42 μg m^−3^ in winter, accounting for 57.0% and 50.3% of PM_2.5_, respectively. Sulfate, nitrate, and ammonium comprised 82.0% and 76.4% of WSIIs in summer and winter, with sulfate dominating in summer and nitrate in winter. Ammonium sulfate and ammonium nitrate were the principal secondary forms, and nighttime conditions favored nitrate formation. Source analyses indicated stronger secondary inorganic aerosol and marine influences in summer, whereas nitrate-rich aerosol, biomass burning, mineral dust, and regional transport were more important in winter. Historical observations from 2009 to 2026 showed substantial declines in PM_2.5_, secondary inorganic ions, and gaseous precursors, with the control challenge shifting after 2019 from sulfate to wintertime nitrate. Coordinated NO_x_–NH_3_ controls are needed, and this compositional shift may have implications for health-relevant PM_2.5_ properties.

## 1. Introduction

Over the past two decades, rapid industrialization, urban expansion, and increasing energy consumption have imposed severe environmental pressures on China, with fine particulate matter (PM_2.5_) pollution becoming one of the country’s most prominent air-quality concerns [1,2]. Water-soluble inorganic ions (WSIIs) constitute a major fraction of PM_2.5_ in many Chinese cities and strongly affect aerosol mass, hygroscopicity, acidity, visibility, and regional climate [3,4]. Among them, sulfate (SO_4_^2−^), nitrate (NO_3_^−^), and ammonium (NH_4_^+^), collectively referred to as SNA, generally dominate the ionic composition [5]. Their abundance is controlled by the emissions and atmospheric transformation of SO_2_, NO_x_, and NH_3_, together with oxidant levels, aerosol liquid water, temperature, relative humidity, and gas–particle partitioning. Accordingly, numerous field and modeling studies have investigated the concentrations, sources, chemical forms, formation pathways, and meteorological controls of WSIIs across China [6,7,8]. These studies have established that SNA are largely produced through atmospheric oxidation followed by neutralization with NH_3_, while combustion emissions, vehicle exhaust, agricultural activities, biomass burning, mineral dust, and regional transport can contribute directly or indirectly to the observed ionic burden [5,9]. This accumulated knowledge has provided an essential scientific basis for identifying major precursor sources and designing particulate-matter control strategies. Informed by this scientific evidence, the Chinese government progressively introduced increasingly stringent clean-air measures, including industrial emission controls, energy-structure adjustments, coal-use reductions, the promotion of cleaner residential fuels, and strengthened vehicle-emission management. These actions substantially reduced anthropogenic emissions, particularly SO_2_, and produced pronounced declines in PM_2.5_ and sulfate concentrations [1,2,10].

However, the chemical composition of PM_2.5_ has evolved substantially during the air-quality improvement process. Multi-year observations across China have consistently shown that sulfate declined more rapidly than nitrate, resulting in increases in both the nitrate fraction and the NO_3_^−^/SO_4_^2−^ ratio and driving a transition from sulfate-dominated to nitrate-dominated aerosol pollution [6,9,11]. Historical observations in Nanchang reveal a similar two-stage evolution. SO_4_^2−^ concentrations decreased rapidly from 2009 to 2019, but the decline slowed markedly after 2019, whereas NO_3_^−^ concentrations remained comparatively stable or increased and the NO_3_^−^/SO_4_^2−^ ratio continued to rise [12,13,14,15,16,17,18,19]. During the same period, reductions in PM_2.5_ concentrations became slower and more variable, with occasional rebounds being particularly evident in winter. Changes in gaseous precursor concentrations, atmospheric oxidation conditions, and regional transport may collectively regulate the composition and abundance of WSIIs. However, the mechanisms driving these recently emerging pollution characteristics remain under debate. Identifying these drivers is therefore essential for developing the next stage of PM_2.5_ control strategies and evaluating the effectiveness of future mitigation measures.

Nanchang, a rapidly developing city in central China, experiences moderate but persistent PM_2.5_ pollution and provides a representative setting for investigating changing urban aerosols outside China’s most intensively studied megacity clusters. Previous measurements have shown that WSIIs constitute a substantial fraction of PM_2.5_ in Nanchang and are strongly influenced by secondary formation, vehicle emissions, coal combustion, biomass burning, mineral dust, and regional transport [12,13,14]. Nevertheless, the present-day seasonal and diurnal characteristics of WSIIs, their formation pathways and controlling factors, and their long-term evolution remain insufficiently resolved. In this study, daytime and nighttime PM_2.5_ samples collected in Nanchang during summer 2025 and winter 2026 were analyzed for nine WSIIs. Ion-equivalent relationships, correlation analysis, redundancy analysis, positive matrix factorization, fire-hotspot data, and air-mass backward trajectories were integrated to characterize ionic composition, formation pathways, environmental controls, and seasonal variations in sources. Historical observations from the past two decades were also compiled to evaluate long-term changes in PM_2.5_, WSIIs, and gaseous precursors and to identify the dominant drivers during different pollution-control stages. The results provide a mechanistic basis for understanding the recent transition in urban WSII pollution and for developing targeted strategies to mitigate wintertime nitrate pollution and achieve further improvements in PM_2.5_ air quality in central China.

## 2. Materials and Methods

### 2.1. Study Area, Sampling Site, and Sample Collection

**Figure 1:**
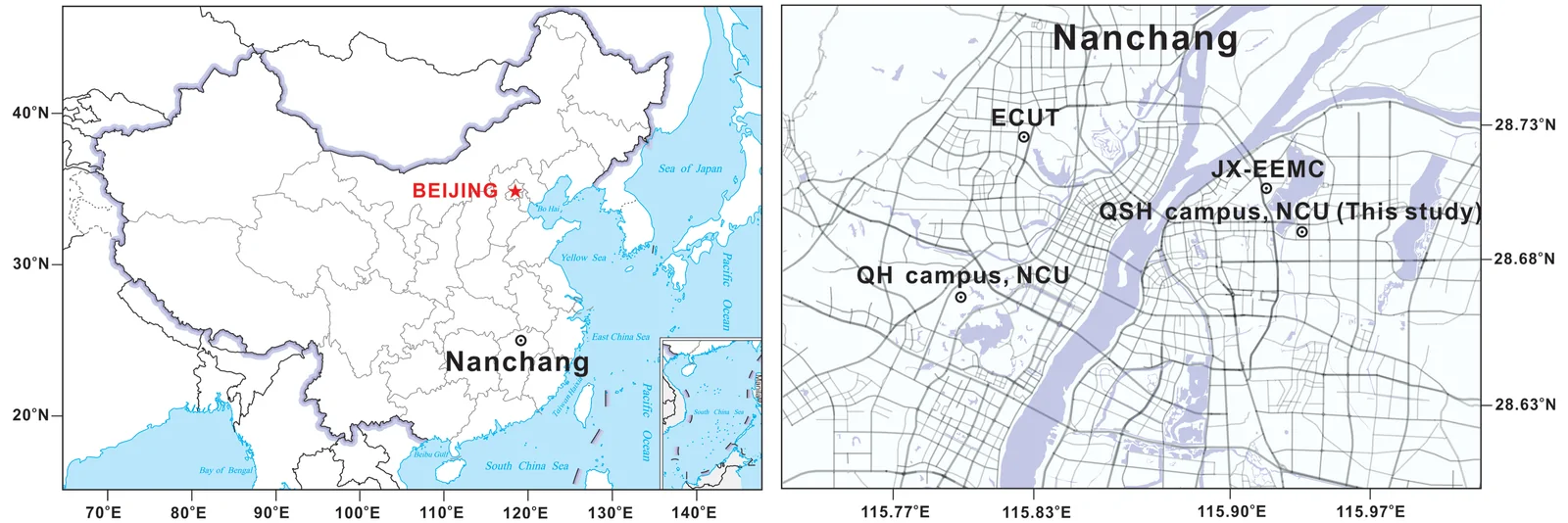
Location of the sampling site in this study, together with the sampling periods and corresponding locations of previously published historical datasets on water-soluble inorganic ions (WSIIs) in Nanchang [12,13,14,15,16,17,18,19]. Abbreviations: ECUT, East China University of Technology; NCU, Nanchang University; QSH Campus, Qingshanhu Campus of NCU; QH Campus, Qianhu Campus of NCU; JX-EEMC, Jiangxi Ecological and Environmental Monitoring Center. The base map of China (**left**) was modified from the China Standard Map Service System (http://bzdt.ch.mnr.gov.cn, accessed on 5 July 2026), and the map showing the sampling station locations within Nanchang (**right**) was adapted from Mapbox Studio (https://www.mapbox.com/, accessed on 8 July 2026). Both maps were further annotated by the authors.

Nanchang, the capital of Jiangxi Province in central China, is located between 115.45° and 116.18° E and 28.15° and 29.18° N. The PM_2.5_ sampling site was situated on the Qingshanhu Campus of Nanchang University (Figure 1). A high-volume sampler equipped with a PM_2.5_ size-selective inlet was installed on the rooftop of an 11-story building, approximately 30 m above ground level. The sampler was unobstructed by nearby buildings or other structures. The surrounding area is characterized by mixed land use, including educational, commercial, residential, and transportation-related activities.

The sampler was operated at a constant flow rate of 1.05 m^3^ min^−1^, and particles were collected on Whatman quartz-fiber filters measuring 20.3 × 25.4 cm (8 × 10 in.). Two consecutive 12-h samples were collected each day, with daytime and nighttime sampling conducted from 09:00 to 21:00 and from 21:00 to 09:00 the following day, respectively. Sampling was conducted from 1–15 July 2025 (summer) and 23 January–6 February 2026 (winter). Of the 60 samples originally scheduled, 58 valid samples were collected, including 30 in summer (15 daytime and 15 nighttime) and 28 in winter (14 daytime and 14 nighttime); one pair of winter daytime and nighttime samples could not be collected because of a power outage.

The locations of previous WSII studies in Nanchang are shown in Figure 1, with additional information on sampling periods, analytical methods, and data sources provided in Table S1. For historical data obtained from unpublished Master’s theses, the original theses were reviewed to verify the sampling site and period, sampling and analytical methods, and reported WSII data before inclusion. Only datasets for which the relevant methodological information and concentration data could be clearly identified were included in the historical comparison.

### 2.2. Analysis of WSIIs and Quality Control

Briefly, aliquots of the quartz-fiber filters were extracted with 50 mL of ultrapure water by ultrasonication, shaking, and centrifugation. The extracts were subsequently filtered and analyzed using a Thermo Scientific Dionex Aquion ion chromatograph (Waltham, MA, USA). Quality assurance and quality control (QA/QC) procedures were implemented throughout the analysis. Calibration curves were established using standard solutions, with coefficients of determination (R^2^) > 0.999. Method detection limits (MDLs) were determined using seven independently prepared low-level spiked blank quartz-fiber filters processed through the complete analytical procedure. The MDL for each ion was calculated as MDL = *t*_(*n*−1, 0.99)_ × *s*, where *s* is the standard deviation of the seven replicate measurements, *n* = 7, and *t*_(6, 0.99)_ = 3.143. The resulting species-specific MDLs are provided in Table S2. Target ion concentrations in the blank filters were below their corresponding MDLs; therefore, no blank correction was applied. Spike-recovery tests were also performed, with recoveries ranging from 92% to 109% for the target ions, indicating acceptable analytical recovery. The relative standard deviations (RSDs) of replicate analyses were <5%.

### 2.3. Meteorological and Air Quality Data Collection

The PM_2.5_ sampling site was located adjacent to a national ambient air quality monitoring station in Nanchang. Hourly PM_2.5_, gaseous pollutant (SO_2_, NO, NO_2_, CO, and O_3_), and meteorological data were obtained from the station during the sampling campaigns. Gaseous pollutant concentrations were measured using analyzers manufactured by Thermo Fisher Scientific Inc. (Waltham, MA, USA) in accordance with the Chinese national standard HJ 654-2013 [20]. Meteorological parameters, including temperature, relative humidity, atmospheric pressure, wind speed, and wind direction, were continuously monitored using an LMD-S4 ultrasonic meteorological sensor (Lanende Intelligent Technology, Weifang, China). For each 12-h sampling period, mean values were calculated from the corresponding hourly observations, except for wind speed and direction, which were retained at hourly resolution. Detailed meteorological and air quality conditions are presented in Text S1 and Figure S1.

### 2.4. Calculation of the Sulfur and Nitrogen Oxidation Ratios

The sulfur oxidation ratio (SOR) and nitrogen oxidation ratio (NOR) are commonly used as indicators of the atmospheric oxidation and secondary formation of particulate sulfate (SO_4_^2−^) and nitrate (NO_3_^−^) from their gaseous precursors, SO_2_ and NO_2_ respectively. SOR and NOR were calculated as follows:

(1) $$\mathrm{SOR}\;={\;\mathrm{SO}}_{4}^{2-}/\left({\mathrm{SO}}_{4}^{2-}\;+\;{\mathrm{SO}}_{2}\right)$$

(2) $$\mathrm{NOR}={\mathrm{NO}}_{3}^{-}/\left({\mathrm{NO}}_{3}^{-}+{\;\mathrm{NO}}_{2}\right)$$

where the SO_4_^2−^, SO_2_, NO_3_^−^ and NO_2_ are the molar concentrations (μmol·m^−3^) in PM_2.5_ and gas phase

### 2.5. Statistical Analyses

Spearman’s rank correlation analysis was used to assess correlations between variables. To account for multiple pairwise comparisons, the resulting *p* values were adjusted using the Benjamini–Hochberg false discovery rate (BH-FDR) procedure. The correction was applied separately to all unique pairwise correlations within each seasonal correlation analysis. Statistical significance for the correlation analysis was determined based on FDR-adjusted *p* values (*p*_adj_ < 0.05). Normality was evaluated using the Shapiro–Wilk test. For comparisons between two independent groups, a two-tailed Welch’s independent-samples t-test was used when both groups were normally distributed; otherwise, the Mann–Whitney U test was applied. For paired comparisons, a two-tailed paired-samples t-test was used when the paired differences were normally distributed; otherwise, the Wilcoxon signed-rank test was applied. Ordinary least-squares (OLS) regression was used for linear fitting. Statistical analyses were performed using Origin 2026.

Redundancy analysis (RDA) was performed using Canoco 5.0 to evaluate the associations of gaseous pollutants and meteorological parameters with SOR and NOR. SOR and NOR were treated as response variables, whereas gaseous pollutant concentrations and meteorological parameters were treated as explanatory variables. Explanatory variables were screened using forward selection, and statistical significance was assessed using Monte Carlo permutation tests with 499 unrestricted permutations. A *p* value < 0.05 was considered statistically significant. The percentage contribution of each explanatory variable was used to characterize its relative association with variations in SOR and NOR. A detailed description of the basic principles of RDA and the interpretation of the canonical axes and RDA biplots is provided in Text S2 of the Supplementary Material.

### 2.6. Positive Matrix Factorization, Backward Trajectory Analysis, and Active Fire Data

US EPA PMF 5.0 was used to apportion WSII sources and quantify their contributions. A total of 58 samples were included in the PMF analysis. The measured ion concentrations and corresponding uncertainties were used as model inputs, with uncertainties calculated from the measured concentrations, method detection limits (MDLs), and analytical error fractions. The stability and uncertainty of the selected solution were evaluated using bootstrap (BS) and displacement (DISP) analyses. Detailed uncertainty calculations, factor selection, and model performance and stability evaluations are provided in Supplementary Text S3. Source contribution concentrations and percentage contributions were calculated for individual samples, and arithmetic averages were used to represent each sampling period.

Seventy-two-hour backward air-mass trajectories initialized at 500 m above ground level were simulated twice daily at 09:00 and 21:00, corresponding to the daytime and nighttime sampling periods, respectively, using NOAA meteorological data and the open-source MeteoInfo software (version 4.1.2). Individual trajectories were overlaid to characterize air-mass transport pathways and potential source regions, and no trajectory cluster analysis was performed. Near-real-time active fire hotspots during the sampling campaigns were obtained from the NASA FIRMS VIIRS 375 m active fire product, with only nominal- and high-confidence detections retained.

## 3. Results and Discussion

### 3.1. Pollution Characteristics of WSIIs

**Figure 2:**
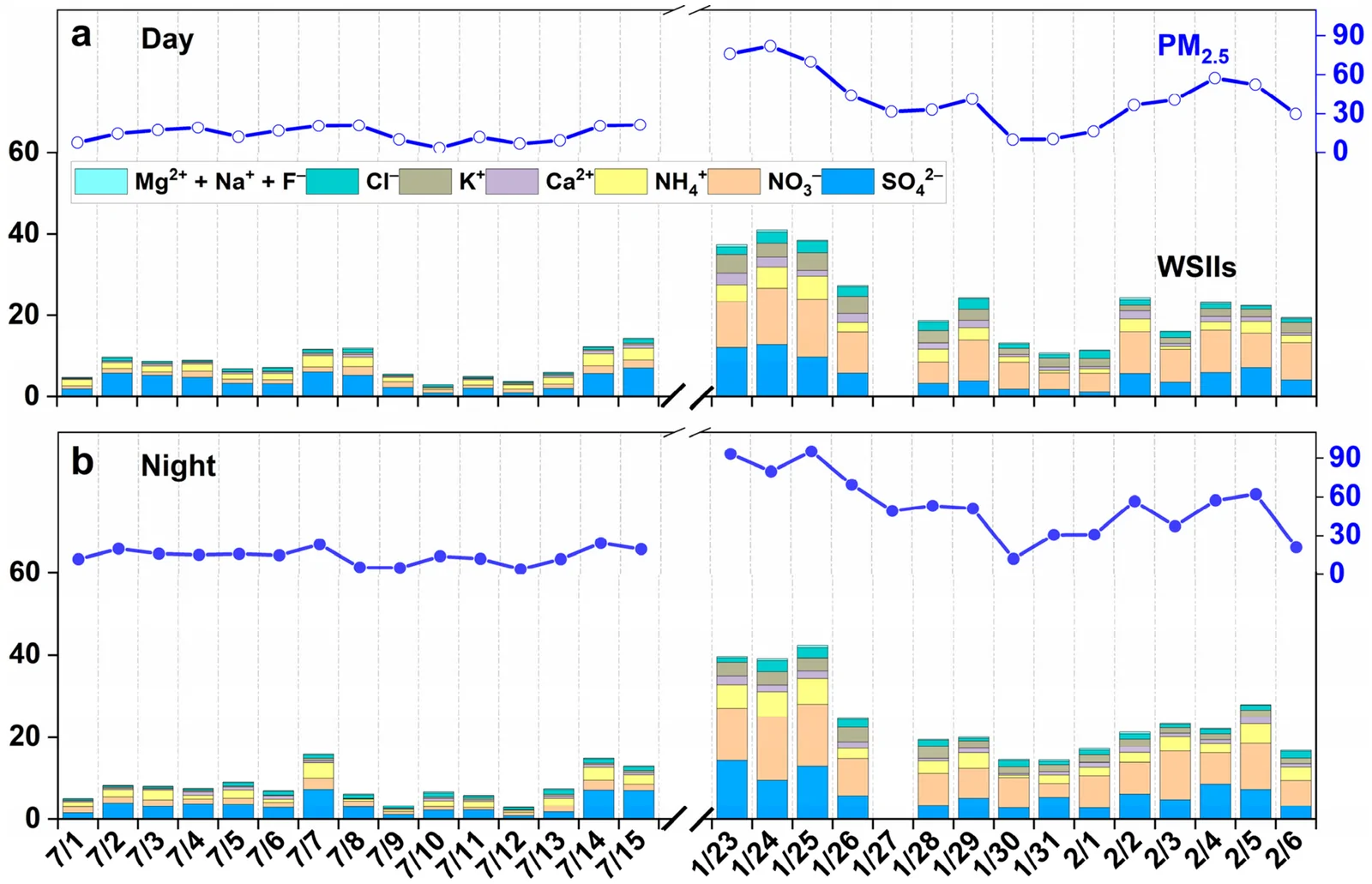
Temporal variations in WSIIs and PM_2.5_ concentrations during sampling (July 2025, January–February 2026); all concentrations in μg m^−3^.

Nine WSIIs were detected during both the summer and winter sampling periods (Figure 2). The total WSIIs concentrations ranged from 2.93 to 15.85 µg m^−3^ in summer, with a mean concentration of 8.01 ± 3.58 µg m^−3^, and from 10.83 to 42.38 µg m^−3^ in winter, with a mean concentration of 23.97 ± 9.42 µg m^−3^. Correspondingly, PM_2.5_ concentrations ranged from 3.42 to 24.17 µg m^−3^ in summer (14.06 ± 5.90 µg m^−3^) and from 9.78 to 95.25 µg m^−3^ in winter (47.62 ± 23.81 µg m^−3^). WSIIs accounted for 57.0% and 50.3% of the PM_2.5_ mass in summer and winter, respectively. The concentrations of both PM_2.5_ and WSIIs were significantly higher in winter than in summer (*p* < 0.05). Moreover, the temporal variation in WSIIs closely paralleled that of PM_2.5_, indicating that WSIIs were major contributors to PM_2.5_ pollution [21,22]. In summer, the daytime concentrations of PM_2.5_ and WSIIs were 13.99 ± 5.85 and 7.99 ± 3.47 µg m^−3^, respectively, which were comparable to their nighttime concentrations of 14.12 ± 6.16 µg m^−3^ and 8.03 ± 3.81 µg m^−3^, respectively. In winter, the nighttime concentrations of PM_2.5_ and WSIIs were 53.23 ± 24.61 and 24.49 ± 9.39 µg m^−3^, respectively, compared with the corresponding daytime concentrations of 42.01 ± 22.38 and 23.46 ± 9.78 µg m^−3^. Paired comparisons showed that the diurnal difference in PM_2.5_ was statistically significant (*p* < 0.05), whereas no significant diurnal difference was observed for WSIIs (*p* > 0.05).

In terms of ionic composition, SNA exhibited higher concentrations than the other measured ions (Figure 2), and accounted for 82.0% and 76.4% of the total WSIIs concentrations in summer and winter, respectively. Clear seasonal differences were observed in the relative abundance of the three major ions. In summer, SO_4_^2−^ was the most abundant ion (3.58 ± 2.03 µg m^−3^), followed by NH_4_^+^ (1.66 ± 0.88 µg m^−3^) and NO_3_^−^ (1.32 ± 0.52 µg m^−3^). In winter, NO_3_^−^ was predominant (9.16 ± 3.25 µg m^−3^), followed by SO_4_^2−^ (6.09 ± 3.62 µg m^−3^) and NH_4_^+^ (3.08 ± 1.65 µg m^−3^). The concentrations of Ca^2+^, K^+^, and Cl^−^ were lower than those of the SNA species. In summer, Cl^−^ had the highest concentration among these ions (0.60 ± 0.26 µg m^−3^), followed by Ca^2+^ (0.45 ± 0.22 µg m^−3^) and K^+^ (0.25 ± 0.13 µg m^−3^). By contrast, K^+^ was the most abundant of these ions in winter (2.43 ± 0.98 µg m^−3^), followed by Cl^−^ (1.57 ± 0.70 µg m^−3^) and Ca^2+^ (1.31 ± 0.63 µg m^−3^). Mg^2+^, Na^+^, and F^−^ were present at relatively low concentrations in both seasons. The observed seasonal differences in WSIIs composition may reflect seasonal variations in emission sources, atmospheric formation pathways, and controlling factors [23,24,25].

### 3.2. Ionic Forms and Formation Mechanisms of WSIIs

**Figure 3:**
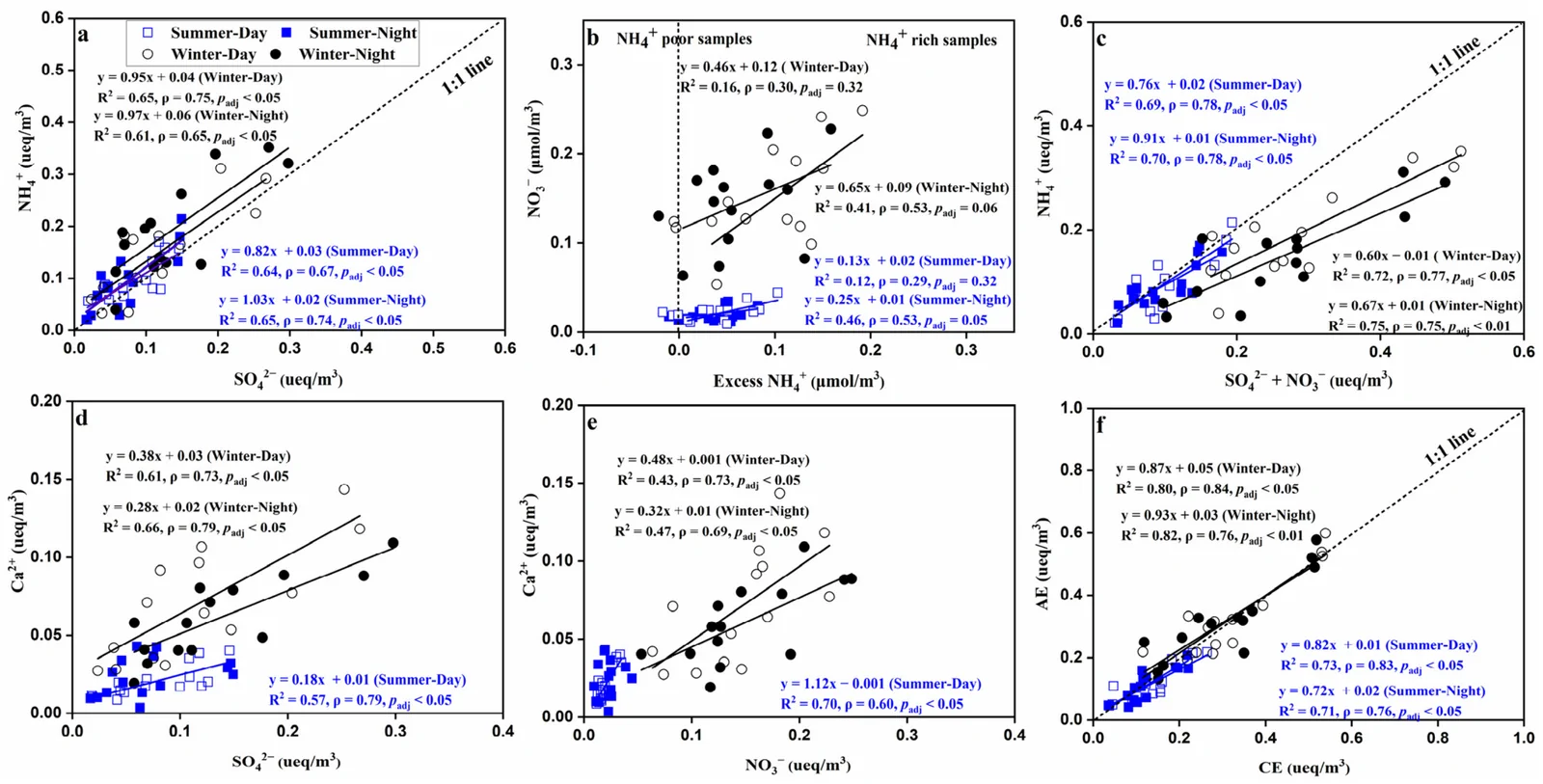
Relationships among selected water-soluble inorganic ions. Molar concentrations were used in panel (**b**), whereas equivalent concentrations were used in panels (**a**,**c**–**f**). Excess [NH_4_^+^] = ([NH_4_^+^]/[SO_4_^2−^] − 1.5) × [SO_4_^2−^], [NH_4_^+^] and [SO_4_^2−^] denote molar concentrations. Solid lines show OLS fits with regression equations and R^2^ values. Spearman’s ρ and BH-FDR-adjusted *p* values (*p*_adj_) are shown; the correlation tests were adjusted together as one family, with *p*_adj_ < 0.05 considered significant.

As noted above, SNA were the dominant components of the measured WSIIs. Their predominant particulate forms were evaluated using equivalent concentration relationships [26]. The equivalent concentrations of SO_4_^2−^ and NH_4_^+^ were significantly correlated in both seasons and during both daytime and nighttime (*p*_adj_ < 0.05), with regression slopes generally greater than 0.5 and close to 1.0 (Figure 3a). These results suggest that neutralization of H_2_SO_4_ by NH_3_ was an important process and that sulfate was predominantly associated with fully neutralized (NH_4_)_2_SO_4_ rather than NH_4_HSO_4_. NH_3_ can also react with HNO_3_ to form particulate NH_4_NO_3_, particularly under NH_3_-rich conditions [4,27]. Previous studies have reported excess atmospheric NH_3_ relative to acidic species in many Chinese cities, including Nanchang [6,28,29]. In the present study, excess NH_4_^+^ was observed in most samples. Although excess NH_4_^+^ and NO_3_^−^ showed moderate positive nighttime associations (ρ = 0.53 in both seasons), these did not remain significant after BH-FDR correction (Figure 3b). Nevertheless, the concurrent nighttime increases in NH_4_^+^ and NO_3_^−^ were consistent with enhanced formation and particle-phase partitioning of NH_4_NO_3_ under lower-temperature and higher-humidity conditions [30,31].

In addition to ammonium salts, SO_4_^2−^ and NO_3_^−^ may also be associated with non-ammonium cations such as Ca^2+^. The regression slopes between the combined equivalent concentration of SO_4_^2−^ and NO_3_^−^ and that of NH_4_^+^ were 0.76 and 0.91 during summer daytime and nighttime, respectively, compared with 0.60 and 0.67 during winter daytime and nighttime (Figure 3c). The lower slopes in winter suggest a greater contribution of non-ammonium cations to sulfate and nitrate neutralization in winter, possibly through the formation of CaSO_4_ and Ca(NO_3_)_2_ [32]. Consistently, Ca^2+^ was correlated with SO_4_^2−^ and NO_3_^−^ in winter (ρ = 0.60–0.79; Figure 3d,e), which may reflect the atmospheric processing of mineral dust by sulfuric and nitric acids [33]. The total anion and cation equivalent concentrations were also closely correlated around the 1:1 line (Figure 3f), indicating approximate charge balance among the measured WSIIs.

### 3.3. Effects of Meteorological Conditions and Gaseous Precursors on WSIIs and the Secondary Conversion of SO_2_ and NO_2_

**Figure 4:**
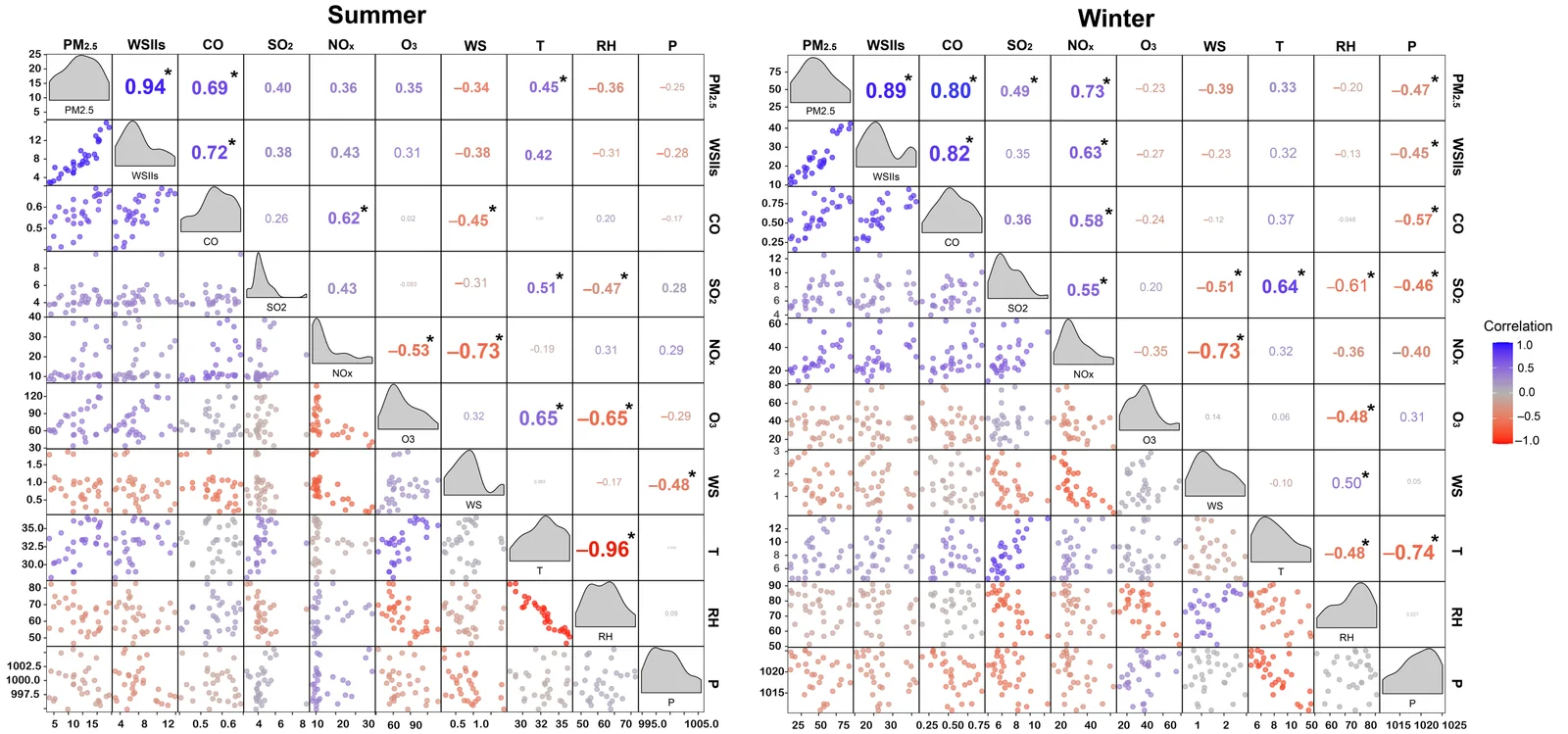
Correlations among meteorological parameters, gaseous pollutants, WSIIs, and PM_2.5_. *p* values from all unique pairwise correlations were adjusted separately for each season using the Benjamini–Hochberg false discovery rate (BH-FDR) procedure. An asterisk (*) indicates a statistically significant correlation based on the FDR-adjusted *p* value (*p*_adj_ < 0.05). Larger numbers indicate higher correlation coefficient values.

Meteorological conditions and gaseous pollutants affect pollutant dispersion, accumulation, and transformation; therefore, their relationships with WSIIs and PM_2.5_ were examined (Figure 4). In summer, WSIIs and PM_2.5_ were positively correlated with CO and temperature but negatively correlated with wind speed. These relationships suggest contributions from combustion-related emissions, enhanced secondary formation at higher temperatures, and pollutant dilution under stronger winds. In winter, WSIIs and PM_2.5_ were positively correlated with CO and NO_x_, indicating that combustion emissions and the co-accumulation of primary pollutants and secondary products contributed to particulate pollution. In contrast, their relationships with meteorological parameters were relatively weak. Overall, the results suggest that both emissions and meteorological conditions influenced summer particulate pollution, whereas winter pollution was more closely associated with combustion-related gaseous pollutants.

**Figure 5:**
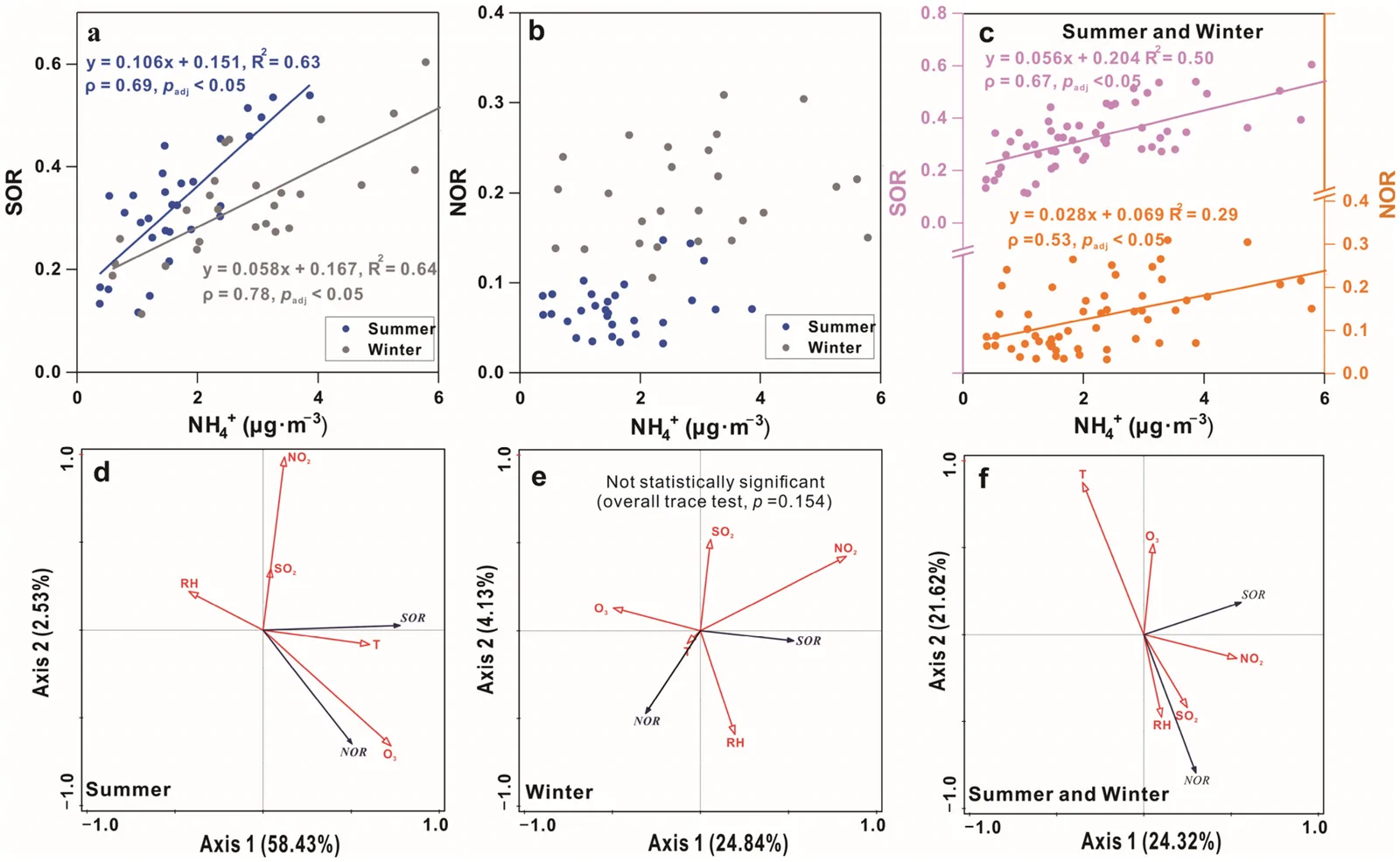
Relationships of particulate NH_4_^+^ with (**a**) SOR and (**b**) NOR for summer and winter samples analyzed separately, and (**c**) with SOR (upper) and NOR (lower) for the pooled dataset. Spearman’s ρ and BH-FDR-adjusted *p* values (*p*_adj_) are shown for panels (**a**–**c**); the tests were adjusted as one family, with *p*_adj_ < 0.05 considered significant. Panels (**d**–**f**) show RDA biplots for (**d**) summer, (**e**) winter, and (**f**) the combined dataset. The winter RDA model was not significant (*p* = 0.154) and was therefore not interpreted. Statistical results of the RDA are summarized in Table S5 of the Supplementary Materials.

The secondary conversion of SO_4_^2−^ and NO_3_^−^ was further examined using the sulfur oxidation ratio (SOR) and nitrogen oxidation ratio (NOR), respectively [34]. Because NH_3_ promotes the neutralization and particle-phase accumulation of sulfate and nitrate [35], the relationships of particulate NH_4_^+^ with SOR and NOR were evaluated by season and for the pooled dataset. After BH-FDR correction, SOR was significantly correlated with NH_4_^+^ in both summer (ρ = 0.69, *p*_adj_ < 0.05) and winter (ρ = 0.78, *p*_adj_ < 0.05) (Figure 5a), whereas NOR showed no significant relationship with NH_4_^+^ within either season (Figure 5b). When both seasons were pooled, NH_4_^+^ was significantly correlated with both SOR (ρ = 0.67, padj < 0.05) and NOR (ρ = 0.53, padj < 0.05) (Figure 5c). These results suggest a more consistent association of particulate ammonium with sulfate conversion than with nitrate conversion at the seasonal scale. Because particulate NH_4_^+^ is not a direct measure of atmospheric NH_3_, these relationships should be interpreted as being consistent with, rather than directly demonstrating, a role of NH_3_ in secondary inorganic aerosol formation [6,11,27,28]. The pooled correlations should also be interpreted cautiously because they may partly reflect seasonal covariance.

RDA was used to quantify the relative associations of SO_2_, NO_2_, and meteorological parameters with SOR and NOR (Table S4 and Figure 5d–f). In summer, higher O_3_ and lower NO_2_ concentrations were associated with increased SOR and NOR, contributing 52.3% and 44.8% of the explained variation, respectively. Higher O_3_ may indicate stronger atmospheric oxidative capacity, whereas the negative association with NO_2_ may reflect its enhanced summertime oxidation and conversion to particulate nitrate. The winter RDA was not statistically significant based on either the overall trace test (*p* = 0.154) or the first canonical axis (*p* = 0.202); therefore, individual variable contributions were not interpreted. For the combined dataset, lower temperature and O_3_, together with higher SO_2_, NO_2_, and relative humidity, were associated with increased SOR and NOR. Temperature contributed most to the explained variation (42.5%), followed by O_3_ (25.3%), NO_2_ (16.9%), and relative humidity (13.4%). These results indicate that temperature, oxidative conditions, precursor abundance, and humidity jointly influence secondary sulfur and nitrogen conversion. However, because the pooled analysis included pronounced seasonal contrasts, these associations may partly reflect covariance between seasons and should be interpreted cautiously.

### 3.4. Major Sources of WSIIs: Evidence from Ionic Ratios, PMF, and Back-Trajectory Analysis

Ionic ratios were first used to qualitatively identify the potential sources of WSIIs. The mean NO_3_^−^/SO_4_^2−^ molar ratio increased from 0.73 ± 0.43 in summer to 2.84 ± 1.22 in winter, suggesting stronger influences of coal-combustion-related secondary sulfate in summer and vehicle-related nitrate formation in winter. The Mg^2+^/Ca^2+^ ratio decreased from 0.18 ± 0.13 to 0.13 ± 0.05, indicating a relatively stronger marine influence in summer and increased mineral-dust input in winter [33,36]. In addition, the Cl^−^/K^+^ ratio declined markedly from 3.78 ± 3.85 in summer to 0.74 ± 0.27 in winter. Together with the elevated winter K^+^ concentrations, this decrease suggests a greater contribution from biomass burning during winter [33,37]. Nevertheless, these ratios should be regarded as qualitative indicators because they may also be affected by source mixing, atmospheric processing, gas–particle partitioning, and differential removal.

PMF analysis resolved five factors (Figure S2). Factor 1 was characterized by a sulfate-rich secondary inorganic aerosol profile, with SO_4_^2−^ and NH_4_^+^ accounting for 57.1% and 25.2% of the factor mass, respectively. Factor 2 was characterized by NO_3_^−^ and K^+^ and was interpreted as a nitrate-rich factor influenced by vehicle emissions and biomass burning. Factor 3 captured 87.6% of the measured F^−^ and was interpreted as an F^−^-related source. Factor 4 was characterized by Ca^2+^, Na^+^, K^+^, and Cl^−^ and represented mixed mineral-dust and marine influences. Factor 5 was characterized by SO_4_^2−^, NO_3_^−^, and NH_4_^+^, which accounted for 42.0%, 29.6%, and 17.7% of its factor mass, respectively, and was interpreted as a mixed SNA-rich secondary inorganic aerosol factor. Factors 1 and 5 both contained substantial secondary inorganic ions, although Factor 1 was more sulfate-rich whereas Factor 5 contained a larger nitrate fraction. Their separation should therefore be regarded as a partitioning of two overlapping secondary-aerosol components rather than as an unequivocal distinction between two specific combustion sources. In summer, Factors 1 and 5 together accounted for approximately 80% of daytime WSIIs and 76% of nighttime WSIIs. Factor 5 individually accounted for approximately 34% during both daytime and nighttime. However, Factor 5 mapped to its corresponding base factor in only 80% of bootstrap runs, compared with 97–98% for Factors 1–4. Therefore, the individual 34% contribution assigned to Factor 5 should be interpreted with caution, and the combined contribution of the two secondary-inorganic-aerosol factors is considered more robust than their individual partitioning. In winter, Factors 1, 2, and 5 were dominant. During daytime, their contributions were 5.36, 9.73, and 3.08 μg m^−3^, corresponding to 19%, 44%, and 15%, respectively; at night, the corresponding contributions were 7.19, 7.97, and 3.50 μg m^−3^, accounting for 28%, 36%, and 18%, respectively. The substantially greater winter contribution from the nitrate-rich Factor 2 was consistent with the seasonal variations in the NO_3_^−^/SO_4_^2−^ and Cl^−^/K^+^ ratios.

**Figure 6:**
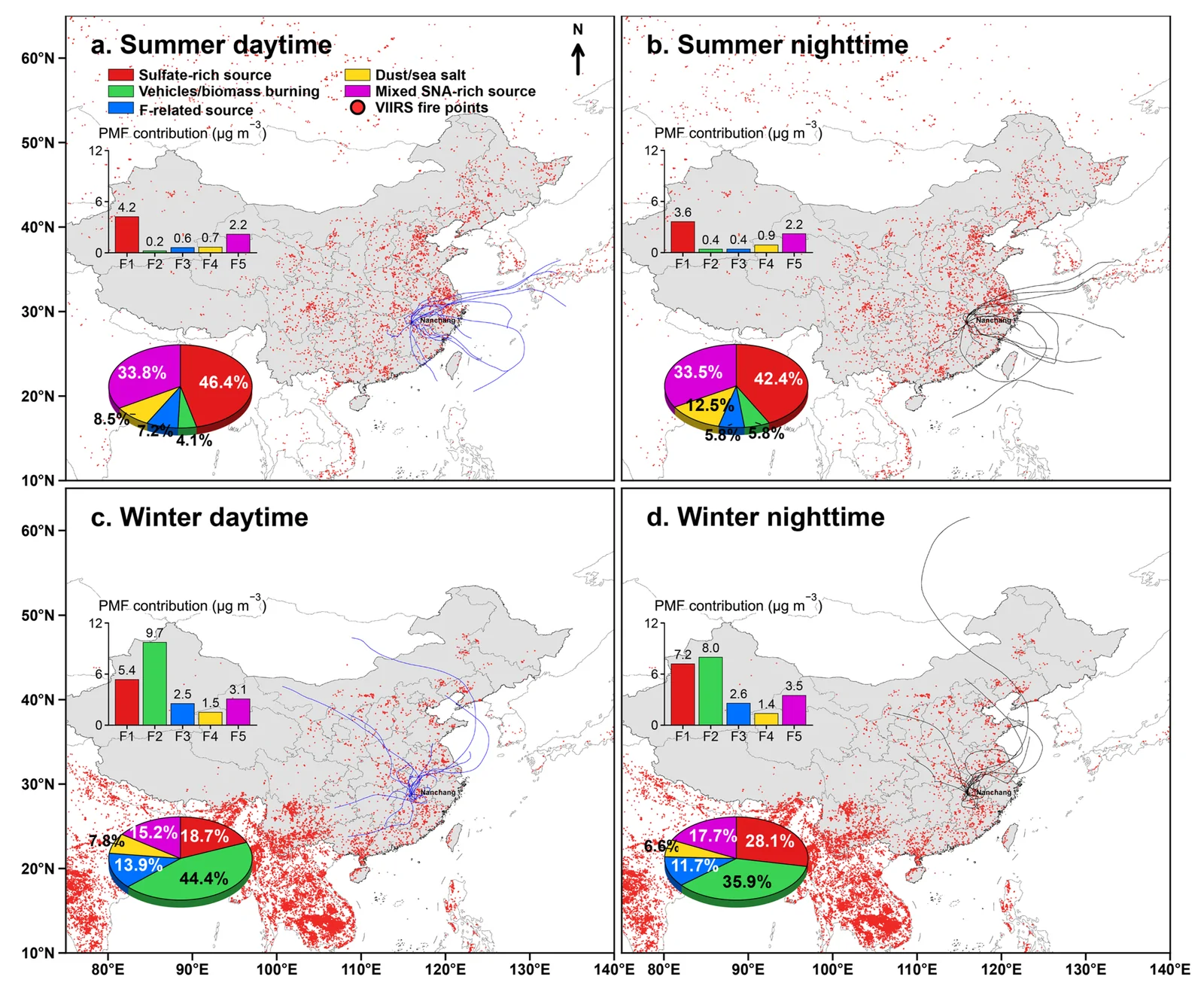
PMF-resolved source contributions interpreted in conjunction with air-mass backward trajectories and spatial distribution of satellite-detected active fires.

Fire-hotspot distributions and air-mass back trajectories further supported these source interpretations (Figure 6). In summer, air masses originated mainly over the ocean and subsequently passed through fire-active regions before reaching the sampling site, indicating combined influences from marine aerosol and regionally transported combustion emissions. Winter fire activity was more extensive, with dense hotspots northwest, northeast, and southwest of the site. Air masses reaching the sampling site from the northeast and southwest passed over several regions with dense fire activity, suggesting a stronger influence of regional combustion emissions in winter. Overall, the combined ionic-ratio, PMF, fire-hotspot, and trajectory evidence indicates clear seasonal differences in WSIIs sources. Summer was more strongly affected by marine aerosol and secondary sulfate, whereas winter showed increased contributions from vehicle-related nitrate, biomass burning, and mineral dust.

### 3.5. Long-Term Evolution of PM_2.5_-Bound WSIIs in Nanchang (2009–2026) and Policy Implications

**Figure 7:**
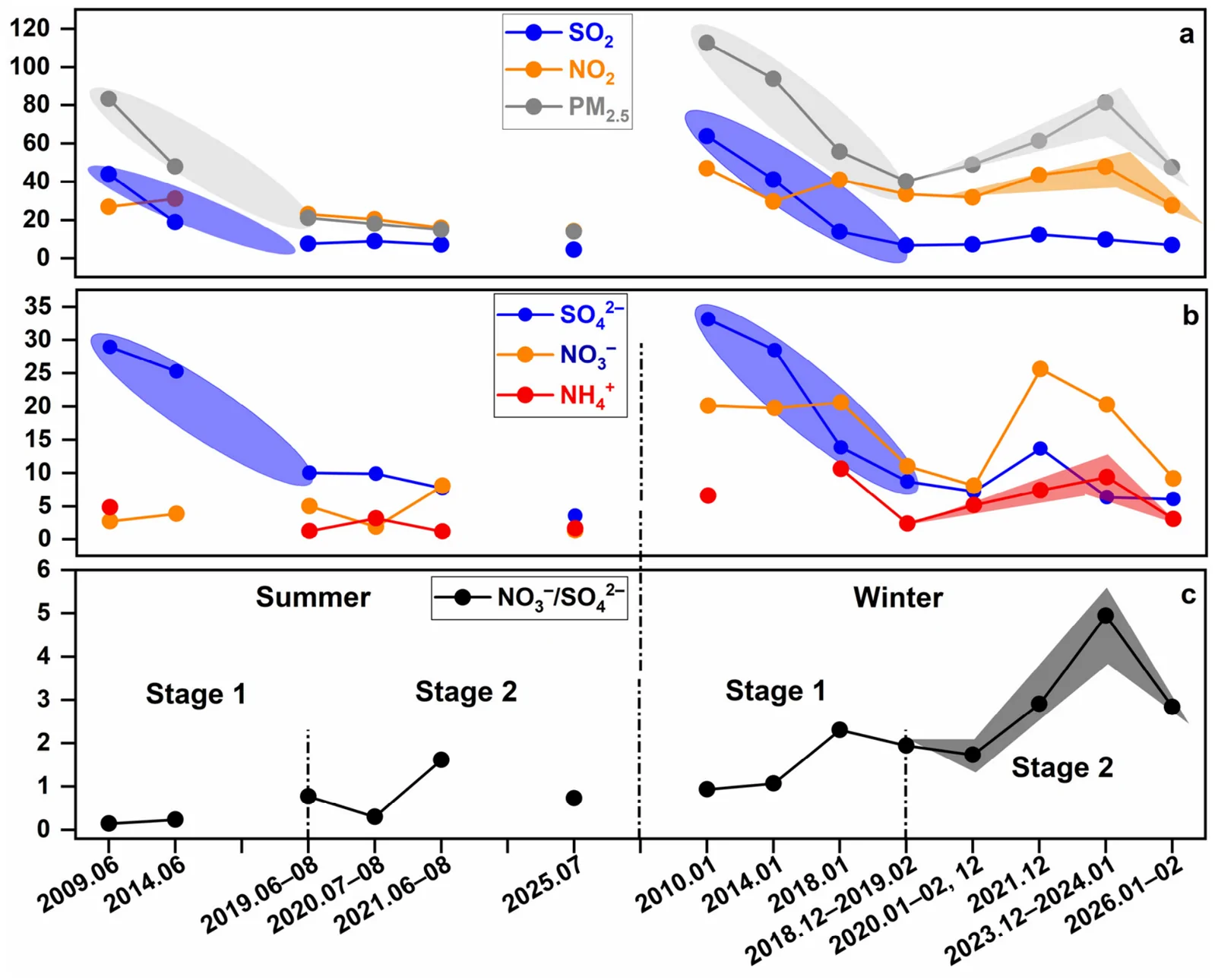
Long-term interannual variations in the concentrations of PM_2.5_, SO_2_, NO_2_, and SNA in Nanchang from 2009 to 2026. (**a**) Annual variations of SO_2_, NO_2_; and PM_2.5_ (**b**) Annual variations of sulfate, nitrate, and ammonium ions. All concentrations are expressed in μg m^−3^. (**c**) Annual variation in the molar ratio of NO_3_^−^/SO_4_^2−^.

Historical observations of WSIIs, gaseous precursors, and PM_2.5_ concentrations in Nanchang from 2009 to 2026 were compiled. Overall, WSIIs, their gaseous precursors, and PM_2.5_ showed substantial long-term declines (Figure 7). SNA, the dominant components of WSIIs, decreased markedly in both seasons. Based on direct comparisons between the earliest and latest available measurements in the compiled datasets, summer SNA was 82% lower in 2025 (6.6 µg m^−3^) than in 2009 (36.5 µg m^−3^), while winter SNA was approximately 70% lower in January 2026 (18.3 µg m^−3^) than in January 2010 (59.9 µg m^−3^) (Figure 7b). The summer 2009 and January 2010 values used to calculate these decreases were outdoor measurements from the rooftop of the Environmental Building at the Qianhu Campus of Nanchang University (approximately 15 m above ground level; Figure 1), both reported in the single unpublished Master’s thesis (Ref. [18]). Similarly, the corresponding endpoint comparisons showed that PM_2.5_ concentrations were 83% lower in summer and 58% lower in winter (Figure 7a). These endpoint comparisons illustrate the magnitude of the long-term changes but should not be interpreted as statistical trend estimates from a continuous time series. The long-term decline in SNA was closely associated with reductions in gaseous precursors, particularly SO_2_. Summer SO_2_ decreased from 44.0 µg m^−3^ in 2009 to 4.5 µg m^−3^ in 2025, while winter SO_2_ declined from 64.0 µg m^−3^ in 2010 to 6.9 µg m^−3^ in 2026, corresponding to reductions of approximately 90% (Figure 7a). These changes coincided with the progressive strengthening of national and municipal air-pollution controls, including major clean-air action plans implemented after 2013. Although the available observations cannot quantify the effects of individual policies, the parallel declines in SO_2_, sulfate, WSIIs, and PM_2.5_ indicate that sustained emission controls were an important driver of long-term air-quality improvement.

**Figure 8:**
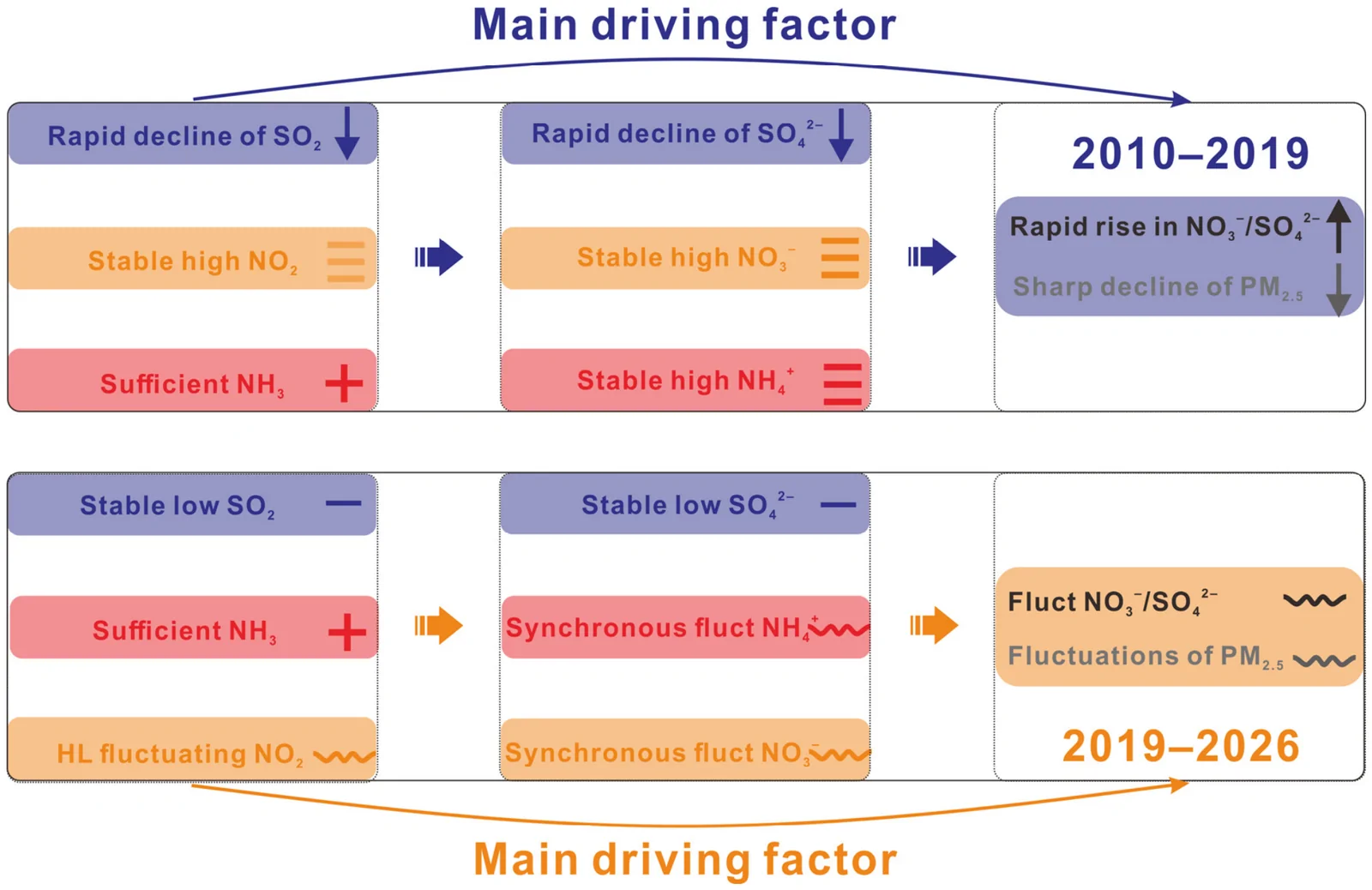
Conceptual diagram of dominant driving factors for long-term PM_2.5_ variations in Nanchang during 2009–2026. Abbreviations: HL, High level.

However, the decreases were not uniform across precursors, ionic species, or time periods, suggesting that the composition and major factors controlling WSIIs evolved over the study period. Accordingly, the long-term evolution of WSIIs can be divided into two stages. This two-stage division is a qualitative interpretation of the evolution observed in the compiled data, rather than a change-point identified through formal statistical analysis. During the first stage, from 2009 to 2019, pollution reduction was dominated by decreases in SO_2_ and sulfate. Summer and winter SO_2_ concentrations fell by 83% and 89%, respectively, while sulfate decreased by 66% in summer and 74% in winter (Figure 7a). In contrast, NO_2_ remained relatively high, and previously reported NH_3_-rich conditions provided sufficient alkaline precursor for nitrate formation [29]. Consequently, nitrate and ammonium declined much less than sulfate (Figure 7b). The increasing NO_3_^−^/SO_4_^2−^ ratio further indicates that PM_2.5_ improvement during this period was driven mainly by SO_2_ control and the resulting reduction in particulate sulfate (Figure 8). During the second stage, from 2019 to 2026, the composition and controlling factors of PM_2.5_ changed. SO_2_ remained at relatively low levels, and the decline in sulfate slowed, especially in winter. Summer sulfate decreased from 10.0 to 3.6 µg m^−3^, whereas winter sulfate declined only from 8.7 to 6.1 µg m^−3^ (Figure 7b). These trends suggest diminishing benefits from further conventional SO_2_ controls. By contrast, NO_2_ remained high and variable, particularly in winter (Figure 7b), while NH_3_ remained sufficiently abundant to promote ammonium nitrate formation [11,29]. Accordingly, the post-2019 variation in PM_2.5_ increasingly paralleled those of NO_2_, nitrate, ammonium, and the NO_3_^−^/SO_4_^2−^ ratio (Figure 7). The December 2023–January 2024 JX-EEMC data were measured using a URG9000D online ambient ion monitor, unlike the filter-based ion chromatography used for the other datasets. As particulate nitrate may volatilize during filter sampling, this methodological difference may partly contribute to the higher nitrate level and NO_3_^−^/SO_4_^2−^ ratio in 2023–2024; nevertheless, the inferred shift is also supported by the precursor data and the present filter-based observations. The dominant precursor constraint therefore appears to have shifted from SO_2_-related sulfate formation to NO_x_-related nitrate formation (Figure 8). Persistent NO_2_ emissions, together with NH_3_-rich conditions, have become a major obstacle to further reductions in wintertime PM_2.5_ (Figure 8).

China’s revised Ambient Air Quality Standards (GB 3095–2026) [38], effective on 1 March 2026, introduced a two-stage implementation schedule. The Class II annual PM_2.5_ limit is 30 µg m^−3^ during 2026–2030 and will be tightened to 25 µg m^−3^ from 2031, while the final Class I limit will be 10 µg m^−3^. Although recent summer PM_2.5_ concentrations in Nanchang have generally remained below 25 µg m^−3^, winter concentrations are still substantially higher and show strong interannual variability. Winter pollution therefore remains the main challenge for compliance with the strengthened annual standard. Future PM_2.5_ control should shift from a predominantly sulfate-oriented strategy toward coordinated NO_x_–NH_3_ management. Further SO_2_ reductions remain beneficial but are unlikely to produce improvements comparable to those achieved before 2019. More targeted and coordinated controls on major NO_x_ and NH_3_ emission sources would suppress ammonium nitrate formation and accumulation, particularly in winter. These measures should be supported by source-specific emission inventories and consideration of meteorological and atmospheric oxidation conditions. Such coordinated control will be essential for overcoming the recent slowdown in PM_2.5_ improvement and achieving sustained compliance with the revised national air-quality standards.

The observed shift from sulfate-dominated to nitrate-dominated secondary inorganic aerosol may also have implications for the health-relevant physicochemical properties of PM_2.5_. Both NO_3_^−^ and SO_4_^2−^ are highly water-soluble, and experimental evidence suggests that these inorganic ions can interact with the pulmonary environment and induce biological responses following inhalation exposure [39,40]. Changes in the relative abundance of nitrate and sulfate may also modify aerosol hygroscopicity and acidity. Under nitrate- and ammonium-rich conditions, nitrate-dominated particles can exhibit enhanced water uptake and higher particle pH than sulfate-rich particles [41,42]. In contrast, highly acidic sulfate-rich aerosols may promote the dissolution of redox-active transition metals, thereby increasing their bioavailability and potentially enhancing particle oxidative potential [43,44]. Toxicological and epidemiological evidence has associated PM_2.5_ nitrate and sulfate with adverse respiratory and cardiovascular effects, although their relative toxicities remain uncertain and may depend on aerosol composition, coexisting pollutants, and exposure conditions [40,44]. Therefore, the increasing importance of nitrate in wintertime PM_2.5_ may alter not only aerosol mass and atmospheric behavior but also health-relevant exposure characteristics. However, because aerosol pH, hygroscopicity, oxidative potential, and biological endpoints were not directly measured in the present study, the relative health risks of nitrate- and sulfate-dominated aerosols cannot be determined from our data and warrant further investigation.

### 3.6. Study Limitations

Several limitations of this study should be acknowledged. First, the field observations were conducted during relatively short 15-day campaigns in summer and winter at a single sampling site, without interannual replication within the present study. Therefore, the observed seasonal differences may not fully represent the spatial and interannual variability of PM_2.5_ and WSIIs across Nanchang. Second, although the BS and DISP diagnostics generally supported the reproducibility and low rotational ambiguity of the five-factor PMF solution, the relatively limited sample size (*n* = 58) and number of input species may constrain source resolution. In particular, Factor 5 mapped to its corresponding base factor in 80% of BS runs, compared with 97–98% for Factors 1–4, and its SNA-rich composition partially overlapped with that of Factor 1. Therefore, the separation, source interpretation, and individual quantitative contribution of Factor 5 should be treated with greater caution than those of the more stable factors. Third, although the Benjamini–Hochberg false discovery rate correction was applied to reduce the potential for false-positive findings arising from multiple pairwise correlations, the correlation analysis identifies statistical associations rather than causal relationships, and the inferred relationships should therefore be interpreted cautiously. Finally, the historical datasets used to characterize long-term variations were obtained from different sampling sites and periods and involved different sampling and analytical protocols. Spatial differences in local emission environments, differences in seasonal and temporal coverage, and methodological inconsistencies among the source studies may affect data comparability. Specifically, the December 2023–January 2024 JX-EEMC dataset was obtained using a URG9000D online ambient ion monitor, whereas the other datasets used filter-based ion chromatography; potential nitrate volatilization during filter sampling may therefore contribute to between-study differences in nitrate concentrations and the NO_3_^−^/SO_4_^2−^ ratio. In addition, some historical data were obtained from unpublished Master’s theses in Chinese. Although these sources were screened for clearly reported sampling information, analytical methods, and WSII data before inclusion, they were not peer-reviewed journal publications, introducing an additional source of uncertainty into the historical comparison. Consequently, the historical datasets should not be regarded as a strictly site-consistent and methodologically uniform continuous time series, but rather as a regional-scale synthesis of the broad long-term evolution of PM_2.5_ and WSIIs in Nanchang. Given this heterogeneity and the discontinuous temporal coverage of the compiled data, formal trend analyses, including the Mann–Kendall test and Sen’s slope estimation, were not applied. The historical comparison was therefore used to characterize broad long-term changes rather than to quantify a statistically defined temporal trend. Future studies incorporating longer-term, multi-site observations with consistent sampling and analytical protocols and larger datasets for source apportionment would help address these limitations.

## 4. Conclusions

This study investigated the seasonal patterns, formation, sources, and long-term trends of PM_2.5_-bound WSIIs in Nanchang. WSIIs averaged 8.01 ± 3.58 µg m^−3^ in summer and 23.97 ± 9.42 µg m^−3^ in winter, accounting for 57.0% and 50.3% of PM_2.5_ mass, respectively. SNA dominated WSIIs, with sulfate prevailing in summer and nitrate in winter, indicating a seasonal shift from sulfate- to nitrate-dominated pollution.

Ion-equivalent relationships suggested that sulfate occurred mainly as fully neutralized ammonium sulfate, while excess NH_4_^+^ and its association with NO_3_^−^ supported ammonium nitrate formation. Stronger nighttime covariation of NH_4_^+^ and NO_3_^−^ indicated that lower temperatures, higher relative humidity, and sufficient NH_3_ favored nitrate formation and particle-phase partitioning.

Meteorological conditions and gaseous pollutants jointly influenced WSIIs accumulation and secondary conversion. Summer pollution was associated with combustion emissions, photochemical processing, and weak dispersion, whereas winter pollution was more closely linked to CO and NO_x_ accumulation. Positive relationships between SOR, NOR, and particulate NH_4_^+^ supported an important role of NH_3_ in secondary aerosol accumulation. Temperature, O_3_, NO_2_ and relative humidity were the primary explanatory variables governing variations in SOR and NOR.

Ionic ratios, PMF, fire-hotspot distributions, and back trajectories revealed clear seasonal source differences. Summer WSIIs were dominated by secondary inorganic aerosol, including a sulfate-rich component and a mixed SNA-rich component, together with marine influence. Winter showed stronger contributions from nitrate-rich secondary aerosol, biomass-burning influence, mineral dust, and regional transport of combustion emissions. Because the mixed SNA-rich Factor 5 showed lower bootstrap stability and partially overlapped chemically with the sulfate-rich Factor 1, its individual quantitative contribution, including the approximately 34% summer estimate, should be interpreted cautiously; the combined secondary-inorganic-aerosol contribution is more robust than the partitioning between these two factors.

Historical observations from 2009 to 2026 showed substantial reductions in PM_2.5_, SNA, and gaseous precursors. SNA decreased by approximately 82% in summer and 70% in winter, while SO_2_ declined by about 90% in both seasons. These percentages are endpoint comparisons based on heterogeneous and discontinuous historical observations and should be interpreted as descriptive indicators of broad long-term change rather than formal statistical trend estimates. During 2009–2019, improvement was driven mainly by SO_2_ control and sulfate reduction. After 2019, sulfate declines slowed, while persistently high NO_2_ and NH_3_-rich conditions increasingly favored nitrate and ammonium formation, especially in winter. The division at 2019 is likewise a qualitative synthesis of the available observations, rather than a statistically identified change-point. Further improvement requires coordinated NO_x_–NH_3_ controls targeting vehicle emissions, biomass burning, fossil-fuel combustion, and agricultural sources, together with stronger regional transport management. The observed shift from sulfate- to nitrate-dominated secondary inorganic aerosol may also modify health-relevant properties of PM_2.5_, highlighting the need for future studies integrating aerosol composition with acidity, hygroscopicity, oxidative potential, and toxicological endpoints.

## Data Availability

The raw data supporting the conclusions of this article will be made available by the authors on request.

## Supplementary Materials

The following supporting information can be downloaded at: https://www.mdpi.com/article/10.3390/toxics14090828/s1, Text S1: Meteorological conditions and air quality; Text S2 Basic principles and interpretation of RDA; Text S3: PMF uncertainty estimation, factor selection, and model performance evaluation; Table S1: Summary of the historical WSII datasets used for the long-term comparison in Nanchang; Table S2: Method detection limits (MDLs) for individual water-soluble inorganic ions; Table S3: Q diagnostics for candidate PMF solutions with different factor numbers; Table S4: Regression diagnostics of the PMF Model; Table S5: RDA statistics for the contributions of meteorological parameters and gaseous pollutants to variations in SOR and NOR; Figure S1: Temporal variations in meteorological parameters and gaseous pollutant concentrations during the sampling period; Figure S2: Source profiles for five factors retrieved via PMF model; Figure S3: Comparison of alternative PMF factor-number solutions: four-factor solution and six-factor solution.

## Abbreviations

The following abbreviations are used in this manuscript:

WSIIs	Water-soluble inorganic ions
SOR	Sulfur oxidation ratio
NOR	Nitrogen oxidation ratio
RDA	Redundancy analysis
PMF	Positive Matrix Factorization
