# Supplementary material for: Seasonal Formation, Sources, and Long-Term Evolution of PM2.5-Bound Water-Soluble Inorganic Ions in Nanchang, China: The Increasing Importance of Wintertime Nitrate
Source: Toxics. 2026 Sep 18;14(9):828. doi: 10.3390/toxics14090828 (PMC13611547; doi:10.3390/toxics14090828)
Supplement: Supplementary file 1 [file toxics-14-00828-s001.zip › toxics-4494519-supplementary.pdf]

Supplementary Materials for

Seasonal Formation, Sources, and Long-Term Evolution of $PM_{2.5}$-Bound Water-Soluble Inorganic Ions in Nanchang, China: The Increasing Importance of Wintertime Nitrate

Wei Guo [1,2,*,†], Zongzhong Ren [3,†], Junkang Wu [3], Kang Du [3], Lichen Deng [4], Yue Qian [4], Yong Luo [5,6], Cheng Liu [1,2], Yue Liang [1,2] and Hao Zhao [1,2]

[1] National Key Laboratory of Uranium Resources Exploration–Mining and Nuclear Remote Sensing, East China University of Technology, Nanchang 330013, China; liuc215@ecut.edu.cn (C.L.); liangyue@ecut.edu.cn (Y.L.); 2024210787@ecut.edu.cn (H.Z.)
[2] Jiangxi Provincial Key Laboratory of Genesis and Remediation of Groundwater Pollution, School of Water Resources and Environmental Engineering, East China University of Technology, Nanchang 330013, China
[3] Nanchang Environmental Engineering Assessment Center, Nanchang 330038, China; ren13732920773@163.com (Z.R.); wjk522050082@163.com (J.W.); paihukang@163.com (K.D.)
[4] Key Laboratory of Climate Change Risk and Meteorological Disaster Prevention of Jiangxi Province, Nanchang 330096, China; 18720068636@163.com (L.D.); qy_yue@126.com (Y.Q.)
[5] Jiangxi Provincial Ecological Environment Monitoring Center, Nanchang 330039, China; u495294416@163.com
[6] State Environmental Protection Key Laboratory of Monitoring for Heavy Metal Pollutants, Changsha 410019, China
* Correspondence: guowei@ecut.edu.cn
† These authors contributed equally to this work.

This file contains Text S1 to S3, Table S1 to S5, Figure S1 to S3. The detailed information is as follows:

1. Text S1. Meteorological conditions and air quality………………………………………….Page 3
2. Text S2. Basic principles and interpretation of RDA …………………………………….Page 3–4
3. Text S3. PMF uncertainty estimation, factor selection, and performance evaluation…….Page 4–6
4. Table S1. Summary of the historical WSII datasets used for the long-term comparison in Nanchang..……………………………………………………………………………...Page 7
5. Table S2. Method detection limits (MDLs) for individual water-soluble inorganic ions……Page 8
6. Table S3. Q diagnostics for candidate PMF solutions with different factor numbers……….Page 9
7. Table S4. Regression diagnostics of the PMF Model………………………………………Page 10
8. Table S5. Redundancy analysis (RDA) statistics for the contributions of meteorological parameters and gaseous pollutants to variations in SOR and NOR………………………..Page 11
9. Figure S1. Temporal variations in meteorological parameters and gaseous pollutant concentrations during the sampling period………………………………………………...Page 12
10. Figure S2. Source profiles for five factors retrieved via PMF model.……….....................Page 13
11. Figure S3. Comparison of alternative PMF factor-number solutions: four-factor solution and six-factor solution.………...Page 14

Text S1. Meteorological conditions and air quality

During the sampling periods, wind speeds ranged from 0.17 to 1.86 m s^{-1} in summer, with a mean of 0.82 ± 0.44 m s^{-1}, and from 0.28 to 2.94 m s^{-1} in winter, with a mean of 1.45 ± 0.72 m s^{-1}. Daytime wind speeds were slightly higher than nighttime values in both seasons. The mean daytime and nighttime wind speeds were 1.04 and 0.61 m s^{-1}, respectively, in summer and 1.53 and 1.39 m s^{-1}, respectively, in winter. Prevailing wind directions also exhibited seasonal differences, with northerly and northwesterly winds dominating in summer and northeasterly winds prevailing in winter. No substantial precipitation occurred during either sampling period. The mean temperature was 32.9 ± 2.4 °C in summer and 8.2 ± 2.7 °C in winter, whereas the mean relative humidity (RH) was 63.7 ± 9.4% and 74.8 ± 11.1%, respectively.

Among the gaseous pollutants, the mean O_3 concentration was higher in summer (76.05 ± 26.44 μg m^{-3}) than in winter (37.38 ± 16.11 μg m^{-3}). In contrast, CO concentrations were comparable between summer (0.57 ± 0.08 mg m^{-3}) and winter (0.56 ± 0.20 mg m^{-3}). The concentrations of SO_2 and NO_x were substantially higher in winter, reaching 6.89 ± 1.93 and 30.41 ± 12.78 μg m^{-3}, respectively, compared with 4.47 ± 1.26 and 15.76 ± 9.15 μg m^{-3} in summer. Pronounced diurnal differences were observed for O_3 and NO_x in summer: the daytime O_3 concentration (96.08 μg m^{-3}) was higher than the nighttime concentration (56.03 μg m^{-3}), whereas the nighttime NO_x concentration (21.37 μg m^{-3}) exceeded the daytime concentration (10.14 μg m^{-3}). No pronounced day–night differences were observed for the other gaseous pollutants (Fig. S1).

Text S2. Basic principles and interpretation of redundancy analysis (RDA)

RDA is a constrained ordination method that combines multivariate multiple linear regression with principal component analysis. In RDA, the response variables are first modeled as linear combinations of the explanatory variables, and the fitted variation is then summarized

along a series of canonical axes. The first canonical axis (RDA1) represents the largest proportion of the constrained variation explained by the explanatory variables, while the second canonical axis (RDA2) represents the largest remaining orthogonal component of the constrained variation. The percentages associated with RDA1 and RDA2 indicate the proportions of the constrained variation represented by the corresponding axes.

In an RDA biplot, the direction and angle between vectors indicate the relationships among variables. Vectors pointing in similar directions indicate positive associations, whereas vectors pointing in opposite directions indicate negative associations; approximately perpendicular vectors indicate weak associations. Longer vectors generally indicate stronger relationships with the ordination pattern represented by the displayed axes. The association between a response variable and an explanatory variable can also be assessed from their relative directions and projections in the ordination space. Statistical significance of the overall RDA model and canonical axes was evaluated using permutation tests.

Text S3. PMF uncertainty estimation, factor selection, and model performance evaluation

The measured concentrations of WSIIs and their corresponding uncertainties were used as inputs to EPA PMF 5.0. For concentrations at or below the species-specific method detection limit (MDL), the uncertainty was calculated as 5/6 × MDL. For concentrations above the MDL, the uncertainty was calculated as：

$$U_{ij} = \left[(EF \times x_{ij})^2 + (0.5 \times MDL_j)^2\right]^{1/2} \quad (S1)$$

where x_{ij} is the measured concentration of species j in sample i, and EF is the analytical error fraction. A uniform error fraction of 0.10 (10%) was applied to all nine ionic species; species-specific error fractions were not used. No additional modeling uncertainty was applied. All nine species were classified as Strong, with signal-to-noise ratios of 9.0, and all samples were retained for modeling. Because the air-equivalent MDLs were substantially lower than most measured

concentrations, the concentration-dependent 10% analytical-error term dominated the uncertainty estimates for most observations.

Candidate solutions containing two to eight factors were initially explored. For each solution containing two to seven factors, 20 base runs were performed. Factor-number selection was based jointly on normalized Q diagnostics, factor-profile interpretability, residual behavior, and uncertainty analyses rather than on the monotonic decrease in Q alone. The $Q_{true}/Q_{expected}$ ratios were 25.14, 18.94, 13.19, 11.21, 11.20, and 13.65 for the two- through seven-factor solutions, respectively, whereas the corresponding $Q_{robust}/Q_{expected}$ ratios were 15.13, 12.53, 9.91, 9.46, 9.81, and 12.91 (Table S3). The eight-factor solution yielded a non-positive Qexpected and was therefore not considered meaningful for normalized-Q comparison.

Increasing the factor number from four to five reduced $Q_{true}/Q_{expected}$ by approximately 15.1% and $Q_{robust}/Q_{expected}$ by 4.6%. In contrast, increasing the number of factors from five to six reduced $Q_{true}/Q_{expected}$ by only 0.07%, while $Q_{robust}/Q_{expected}$ increased from 9.46 to 9.81. The four-factor solution also showed appreciable mixing of chemically distinct tracers. For example, although approximately 89% of F^{-} was assigned to one factor, that factor was dominated in total mass by NO_3^{-}, SO_4^{2-}, and K^{+}, while mineral/sea-salt and secondary species were also distributed among mixed factors (Figure S3a). In the six-factor solution, the additional factor did not yield a comparably distinct new source profile; instead, sulfate, ammonium, Ca^{2+}, Mg^{2+}, Cl^{-}, and other tracers were further redistributed among several mixed factors (Figure S3b). Thus, the five-factor solution represented the most parsimonious compromise between model fit and chemical interpretability. The absolute normalized Q values remained greater than unity; therefore, they were used primarily for comparative factor-number evaluation rather than as evidence of an ideal statistical fit.

For the selected five-factor solution, 20 base runs were performed using a base random seed of 68, and Base Run #8, which had the lowest Q_{robust} (1768.16), was used for subsequent

interpretation. Model reconstruction was evaluated from regressions between observed and PMF-predicted concentrations. The coefficients of determination (R^2) ranged from 0.75 to 0.99, with R^2 ≥0.84 for eight of the nine species (Table S4). The stability of the five-factor solution was further evaluated using bootstrap (BS) and displacement (DISP) analyses. In 100 BS runs, Factors 1–5 mapped to their corresponding base factors in 98%, 97%, 97%, 97%, and 80% of runs, respectively. The DISP analysis returned no errors, the maximum decrease in Q was 0.186, and no factor swaps occurred at dQ_{max} values of 4, 8, 15, or 25. These diagnostics generally support the reproducibility and low rotational ambiguity of the five-factor solution. However, Factor 5 showed lower bootstrap stability than Factors 1–4 and partially overlapped chemically with Factor 1. Therefore, the separation and individual quantitative contribution of Factor 5 are considered less certain, and the combined contribution of the secondary-inorganic-aerosol factors is interpreted more robustly than their individual partitioning.

Table S1. Summary of the historical WSII datasets used for the long-term comparison in Nanchang. Abbreviations: ECUT, East China University of Technology; NCU, Nanchang University; QSH Campus, Qingshanhu Campus of NCU; QH Campus, Qianhu Campus of NCU; JX-EEMC, Jiangxi Ecological and Environmental Monitoring Center. Corresponding citations are provided in the manuscript reference list.

Year	Sampling site	Summer sampling period	Winter sampling period	Analytical method/Instrument	Data source
2009–2010	QH Campus, NCU	Jun 2009	Jan 2010	Ion chromatography/Thermo Scientific Dionex-600	[18]
2014	QH Campus, NCU	Jun 2014	Jan 2014	Ion chromatography/Metrohm IC-861	[16]
2018	ECUT	n.d	Jan 2018	Ion chromatography/Thermo Scientific Dionex Aquion	[13]
2018–2019	QH Campus, NCU	Jun–Aug 2019	Dec 2018–Feb 2019	Ion chromatography/Thermo Scientific Dionex ICS-1100	[19]
2020	ECUT	Jul–Aug 2020	Jan–Feb and Dec 2020	Ion chromatography/Thermo Scientific Dionex Aquion	[15]
2021	QH Campus, NCU	Jun–Aug 2021	Dec 2021	Ion chromatography/Thermo Scientific Dionex ICS-1100	[17]
2023–2024	JX-EEMC	n.d	Dec 2023–Jan 2024	Ion chromatography/Thermo Fisher URG9000D	[14]
2025–2026	QSH Campus, NCU	Jul 2025	Jan 2026	Ion chromatography/Dionex Aquion	This study

Table S2. Method detection limits (MDLs) for individual water-soluble inorganic ions. Extract-based MDLs were converted to atmospheric concentration units using the same filter fraction, extraction volume, and sampled air volume used for ambient concentration calculations.

Ions	SO_4^{2-}	NO_3^-	NH_4^+	Ca^{2+}	Cl^-	Mg^{2+}	Na^+	K^+	F^-
MDL in extract (μg L^{-1})	7.92	15.08	7.26	6.77	4.25	15.67	7.05	12.78	3.10
MDL used for $PM_{2.5}$ and PMF (μg m^{-3})	0.002	0.005	0.002	0.002	0.001	0.005	0.002	0.004	0.001

Table S3. Q diagnostics for candidate PMF solutions with different factor numbers.

Number of factors	Q_{robust}	Q_{true}	$Q_{expected}$	$Q_{true}/Q_{expected}$	$Q_{robust}/Q_{expected}$
2	5870.96	9755.72	388	25.14	15.13
3	4021.6	6080.94	321	18.94	12.53
4	2517.13	3351.44	254	13.19	9.91
5	1768.16	2095.86	187	11.21	9.46
6	1176.75	1344.01	120	11.20	9.81
7	684.041	723.209	53	13.65	12.91
8	229.519	229.519	-14	n.a.	n.a.

Note: For each two- through seven-factor solution, 20 base runs were performed. The eight-factor solution was initially explored but yielded a non-positive $Q_{expected}$; therefore, normalized Q ratios were not interpreted for this solution. The five-factor solution was selected based on the combined evaluation of normalized Q values, factor-profile interpretability, residual behavior, and BS/DISP diagnostics.

Table S4. Observed–predicted regression and scaled-residual diagnostics for the selected five-factor PMF solution.

Species	Intercept	Slope	SEE	r^2	KS *p* Value
SO_4^{2-}	0.06	0.93	0.97	0.90	0.13
NO_3^-	0.19	0.89	1.35	0.90	0.95
NH_4^+	0.06	0.91	0.45	0.90	0.83
Ca^{2+}	0.13	0.71	0.17	0.88	0.98
Mg^{2+}	0.00	1.04	0.00	0.98	0.13
Na^+	0.00	0.94	0.03	0.84	0.02
K^+	0.06	0.90	0.29	0.94	0.95
F^-	0.00	0.96	0.00	0.99	0.01
Cl^-	0.11	0.75	0.31	0.75	0.97

Note: Observed concentrations were used as the independent variable and PMF-predicted concentrations as the dependent variable. SEE denotes the standard error of estimate of the ordinary least-squares regression, and has the same units as concentration. The KS statistic and KS p value are from the Kolmogorov–Smirnov test of the normality of scaled residuals and are not p values for the statistical significance of the observed–predicted regression.

Table S5. Redundancy analysis (RDA) statistics for the contributions of meteorological parameters and gaseous pollutants to variations in SOR and NOR. Gray-shaded cells represent results without statistical significance ($p > 0.05$)

Summary Table					Summer			
Statistic	Axis 1	Axis 2	Axis 3	Axis 4	Name	Explains %	Contribution %	*p*
Eigenvalues	0.5843	0.0253	0.3722	0.0182	O_3	31.9	52.3	0.004
Explained variation (cumulative, %)	58.43	60.96	98.18	100	NO_2	27.3	44.8	0.002
Pseudo-canonical correlation	0.784	0.7157	0	0	RH	1.4	2.4	0.332
Explained fitted variation (cumulative, %)	95.84	100			SO_2	0.1	0.2	0.84
					T	0.2	0.3	0.79
Summary Table					Winter			
Statistic	Axis 1	Axis 2	Axis 3	Axis 4	Name	Explains %	Contribution %	*p*
Eigenvalues	0.2484	0.0413	0.6366	0.0737	NO_2	17.8	61.3	0.022
Explained variation (cumulative, %)	24.84	28.97	92.63	100	RH	6.2	21.5	0.152
Pseudo-canonical correlation	0.5818	0.3941	0	0	SO_2	1.4	4.7	0.56
Explained fitted variation (cumulative, %)	85.73	100			T	2.1	7.2	0.496
					O_3	1.5	5.3	0.538
Summary Table					Summer and Winter			
Statistic	Axis 1	Axis 2	Axis 3	Axis 4	Name	Explains %	Contribution %	*p*
Eigenvalues	0.2432	0.2162	0.4803	0.0602	T	19.5	42.5	0.002
Explained variation (cumulative, %)	24.32	45.94	93.98	100	O_3	11.6	25.3	0.002
Pseudo-canonical correlation	0.5813	0.8782	0	0	NO_2	7.8	16.9	0.014
Explained fitted variation (cumulative, %)	52.94	100			RH	6.2	13.4	0.024
					SO_2	0.9	1.9	0.368

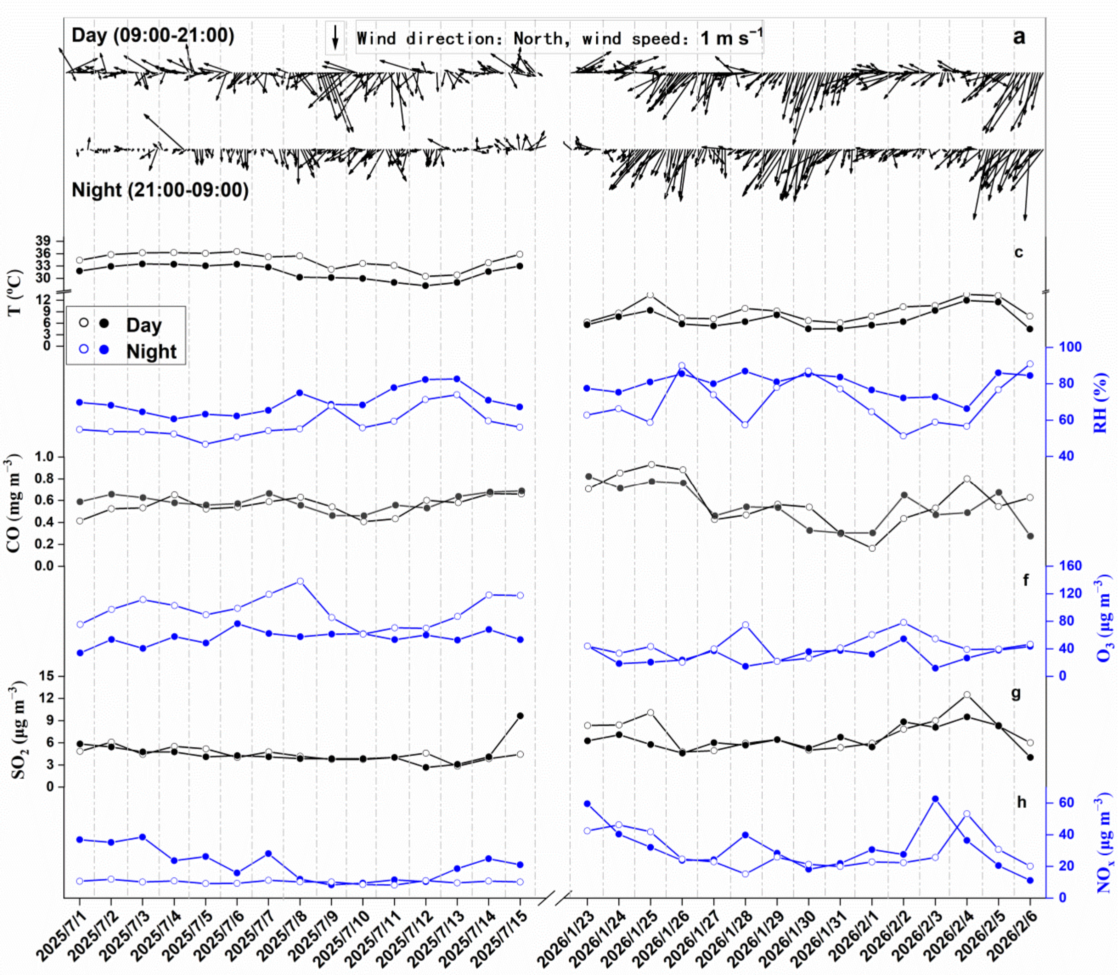

Figure S1. Temporal variations in meteorological parameters and gaseous pollutant concentrations during the sampling period.

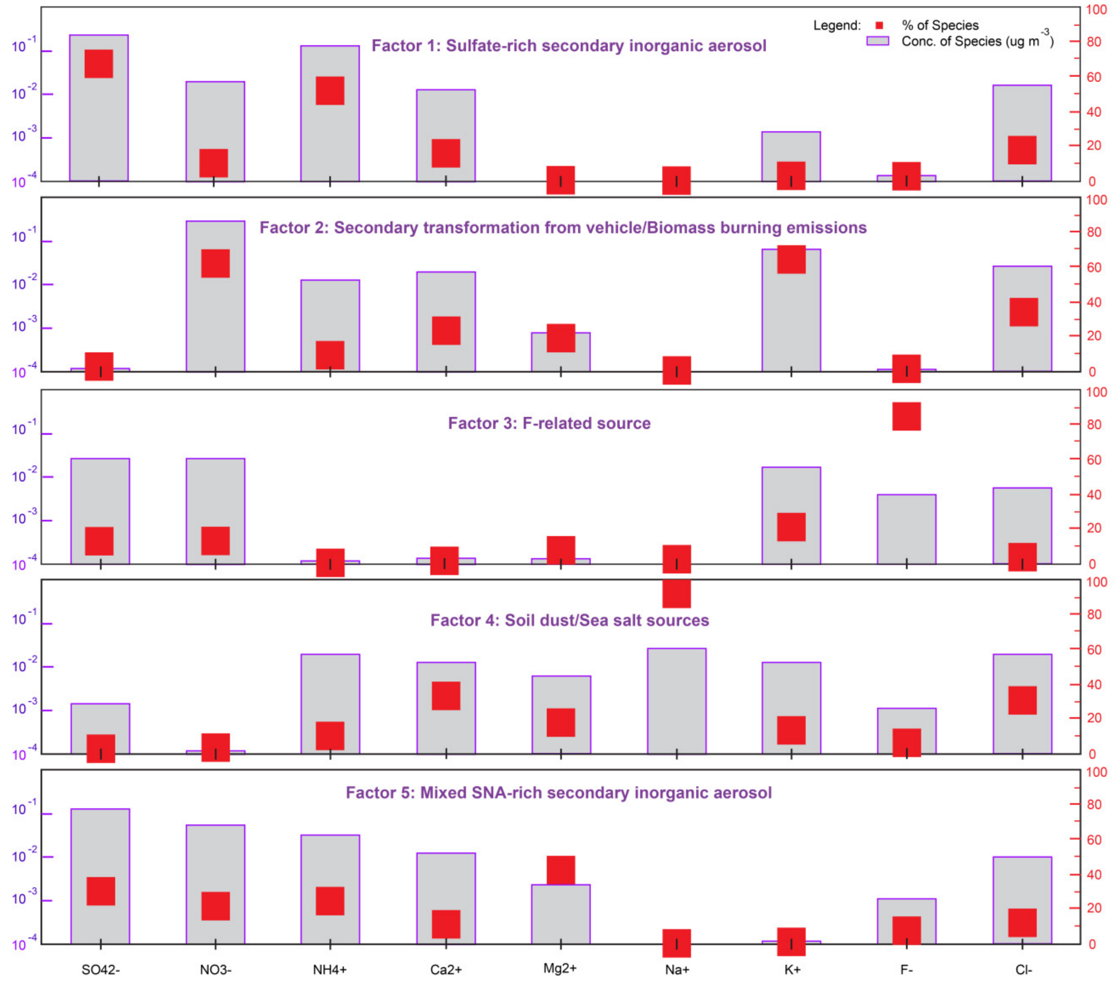

Figure S2. Source profiles for five factors retrieved via PMF model, including absolute ionic concentrations and proportional percentages of total species mass.

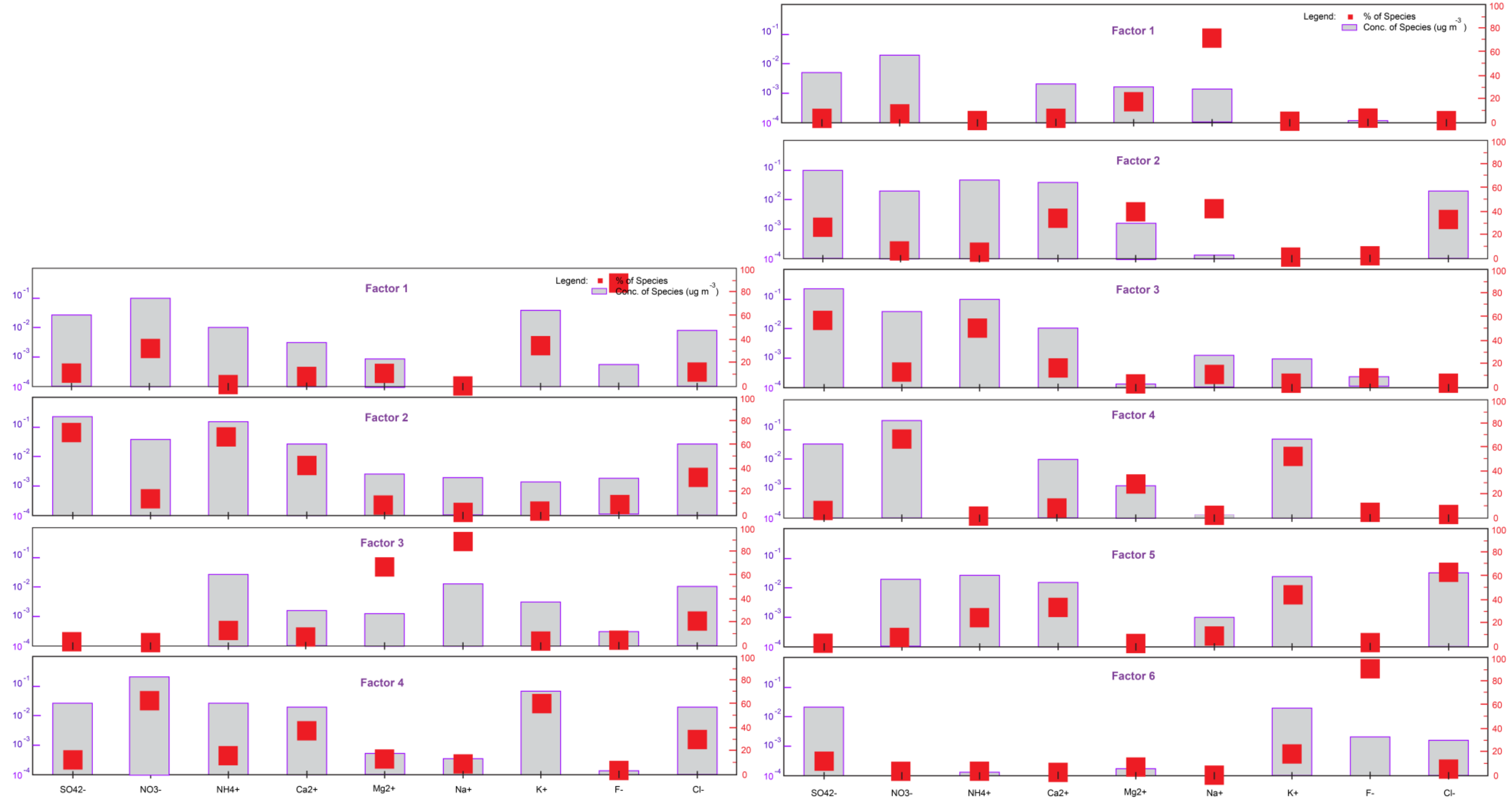

Figure S3. Comparison of alternative PMF factor-number solutions: four-factor solution and six-factor solution. The four-factor solution showed greater mixing of chemically distinct tracers, whereas the six-factor solution produced additional splitting of related species without a meaningful improvement in normalized Q compared with the five-factor solution.
